# Bilateral Frontoparietotemporal Craniectomy for Traumatic Brain Injury: A Case Report

**DOI:** 10.7759/cureus.49410

**Published:** 2023-11-25

**Authors:** Recai Engin, Abdullah Hilmi Marangoz, Fatih Tomakin, Cengiz Çokluk

**Affiliations:** 1 Department of Neurosurgery, Kahramanmaras Necip Fazıl City Hospital, Kahramanmaras, TUR; 2 Department of Neurosurgery, Faculty of Medicine, Ondokuz Mayıs University, Samsun, TUR; 3 Department of Neurological Surgery, Unye State Hospital, Ordu, TUR

**Keywords:** surgical case reports, severe head trauma, pediatric surgery, decompressive craniectomy, traumatic brain injury, kjellberg procedure

## Abstract

There is no conclusive agreement on the optimal approach to managing severe traumatic brain injury. This article details the methodology and outcomes of bilateral frontoparietotemporal decompression surgery performed on a three-year-old patient with severe traumatic brain injury. As the patient had fixed dilated pupils, GCS (Glasgow coma scale) 4, and marked edema in the frontal and parietal regions, the Kjellberg approach was modified, and decompression including part of the parietal bone was performed. The patient was intubated and sedated in the intensive care unit for one week postoperatively. After extubation, the patient had reactive pupils and a GCS of 13. The patient underwent a cranioplasty two months after the trauma, combining the bone grafts placed in the abdomen. The patient was followed for three days after cranioplasty and discharged with a GCS:15 and intact motor examination.

## Introduction

Traumatic brain injury (TBI) is a major public health problem worldwide [1]. TBI is recognised as the leading cause of death in the young population (<45 years) [2]. The incidence of pediatric TBI hospitalization is reported to be 70 cases per 100,000 children [3]. The use of surgical decompression in the form of decompressive craniectomy (unilateral or bilateral) to reduce increased intracranial pressure after TBI has increased significantly over the last 20 years [4]. Studies have shown that surgical decompression is preferable to conservative management in patients with TBI owing to its advantages, such as reducing increased intracranial pressure, increasing cerebral perfusion, preventing secondary brain damage, shortening hospital stay, and reducing morbidity and mortality [2,5,6]. The main goal of this approach is to achieve significant cranial volume expansion. The most important prognostic factors include patient age, comorbidity, initial Glasgow coma scale (GCS) and early surgical intervention [7,8]. Indications for decompressive craniectomy remain controversial. Particularly, there is no consensus on whether surgery should be performed in patients with a GCS of less than five and fixed dilated pupils [9]. Additionally, the technique of craniectomy and the size of the craniectomy are controversial [10].

Bilateral frontotemporal craniectomy (Kjellberg procedure) was popularised by Kjellberg in the 1970s. This method can be used to create space in the frontal lobe, especially in patients with severe TBI. However, it will not be sufficient to decompress the parietal and occipital regions [6].

## Case presentation

A three-year-old male patient was brought to the emergency department approximately 30-45 minutes after an in-vehicle traffic accident. He was intubated, did not open his eyes to a painful stimulus, had no verbal response, and had an abnormal extensor posture. The patient's GCS on admission was four. Pupils were fixed dilated, and there was no light reflex in both pupils. The patient's past medical history was unremarkable. The patient had no other systematic injuries. Haemodynamics were stable, and blood pressure and pulse rate were within normal limits. There were no abnormal blood parameters.

**Figure 1:**
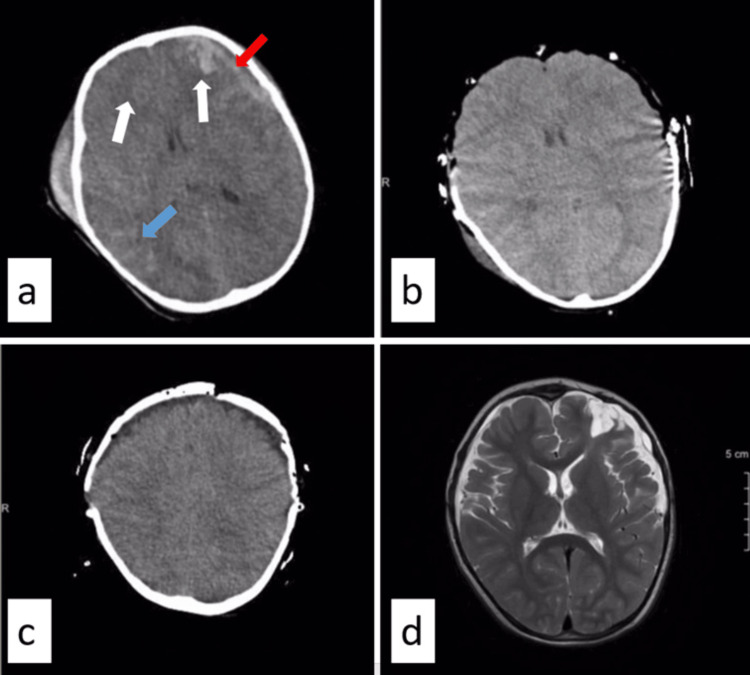
a. Computed tomography image at the time of admission, showing acute subdural haematoma in the left frontotemporal region (red arrow), contusion in the bilateral frontal region (white arrow), and traumatic subarachnoid haemorrhage in the right temporal region (blue arrow). b. Imaging after bilateral frontotemporoparietal decompression. c. Tomography image after cranioplasty with bones removed from the abdomen two months after trauma. d. T2-weighted magnetic resonance imaging six months after trauma. There is a significant subdural effusion on the left.

**Figure 2:**
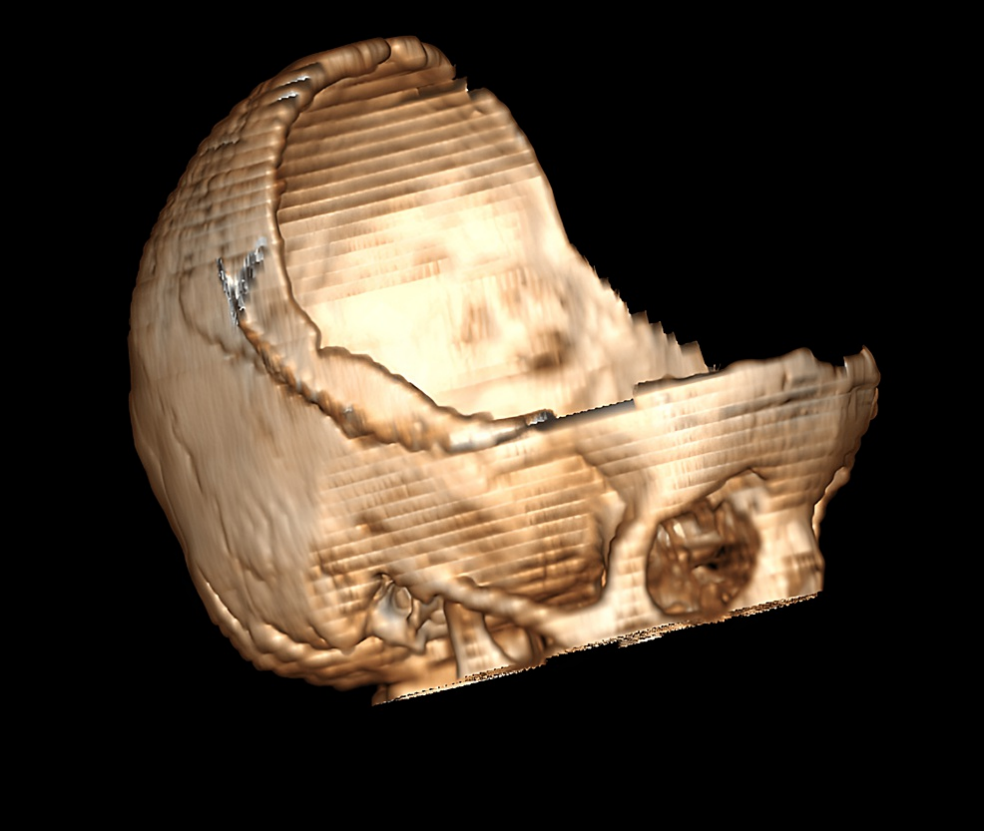
3D animation of postoperative brain CT.

Cranial imaging revealed an acute subdural haematoma approximately 1 cm thick in the left frontotemporal region, contusion, and oedema approximately 2×1 cm in the left frontal lobe and traumatic subarachnoid haemorrhage in the right temporal lobe (Figure 1). For a better understanding of the decompression performed on the patient, a 3D illustration of the postoperative CT scan of the brain is shown in Figure 2.

Emergency decompressive craniectomy was planned. Although the Kjellberg approach was initially considered, it was decided that only frontotemporal decompression would not be efficient, and a wider decompression including part of the parietal bone was preferred. This was the main difference between the surgical technique and the Kjellberg procedure. The operation took about three hours and 30 minutes. The surgical procedure and other details are described in detail in the figures.

The patient was transferred to the paediatric intensive care unit after surgery. The patient was intubated for one week and sedated with midazolam at a dose of 1 mg/kg intravenous infusion. The patient was treated with 3% hypertonic saline 0.5 mL/kg/h intravenous infusion for three days and was monitored in the 30-degree Fowler's position. As there were no cooling devices in our intensive care unit, no treatment such as hypothermia could be applied. 

Sedation was discontinued at the end of one week. On examination one day after discontinuation of sedation, eye opening to verbal stimulation, inappropriate word response, motor examination obeyed commands, and GCS was 13. After two days, the patient's examination showed complete recovery and GCS was 15. Pupils were isochoric, light reflex was +/+, and motor examination was intact. After two weeks in intensive care, the patient was transferred to our neurosurgery ward and discharged after one week. The only medical treatment given was levetiracetam at a dose of 20 mg/kg intravenously two times daily.

After one month of outpatient follow-up, the patient was admitted to the hospital for cranioplasty. The patient was prepared for surgery, and replacement cranioplasty was performed approximately two months after craniectomy using only the bone in the abdominal subcutaneous fat. The patient was followed for three days after cranioplasty and discharged with a GCS:15 and no neurological deficit.

Brain MR imaging performed six months later showed a 1.5 cm non-compressive subdural effusion in the left frontal region (Figure 1). The patient has no further complaints, and no surgical intervention is planned.

Surgical Technique

**Figure 3:**
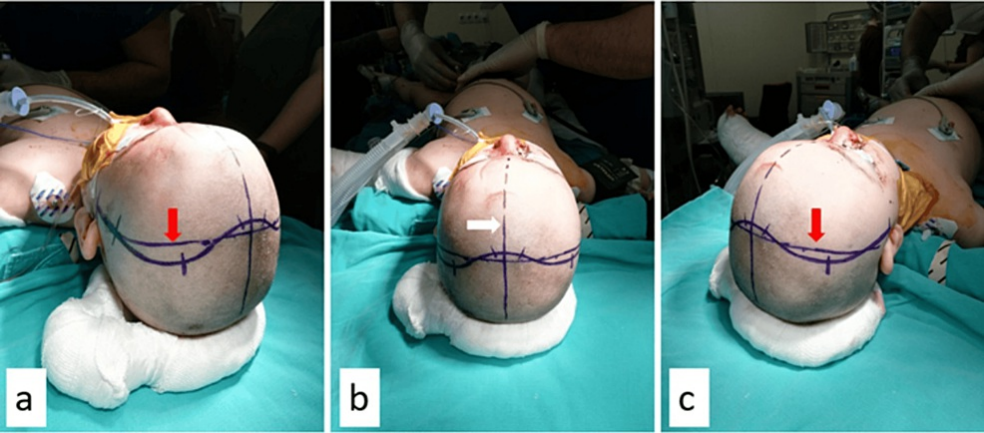
Surgical supine position and proposed incision line. Coronal suture (red arrow) and midline sagittal suture (white arrow). A bicoronal incision was planned to be behind the hairline. The head was supported with surgical drapes under the shoulder, and a slight extension of the head was performed.

**Figure 4:**
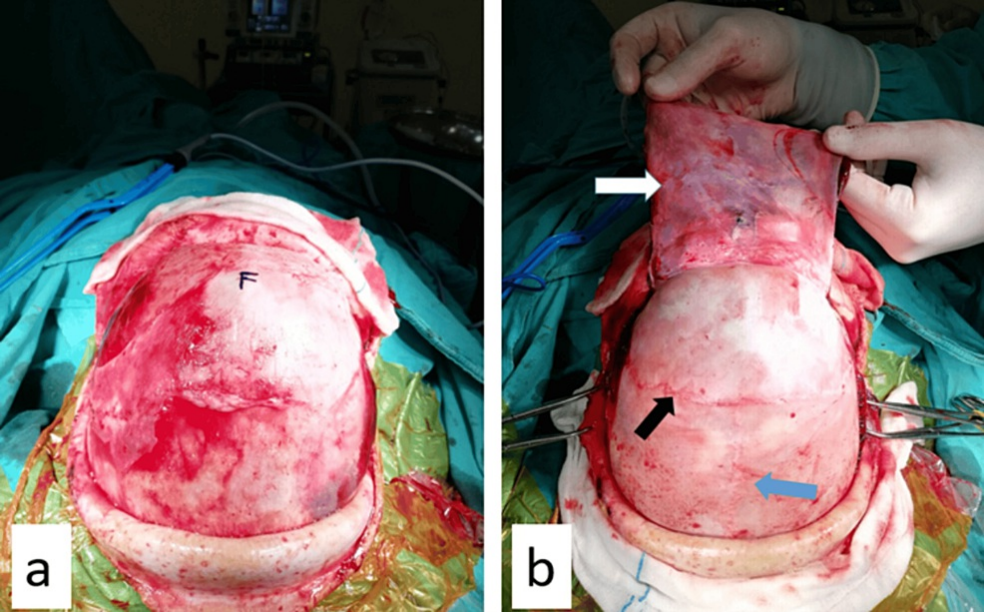
a. Skin subcutaneous scalpel incision was made, and the skin was folded towards the frontal base and parietal, leaving the galea on the cranium. b. The galea was peeled off the periosteum, and the frontal base was removed to be used for duraplasty (white arrow), and coronary suture (black arrow) and sagittal suture (blue arrow) are visible.

**Figure 5:**
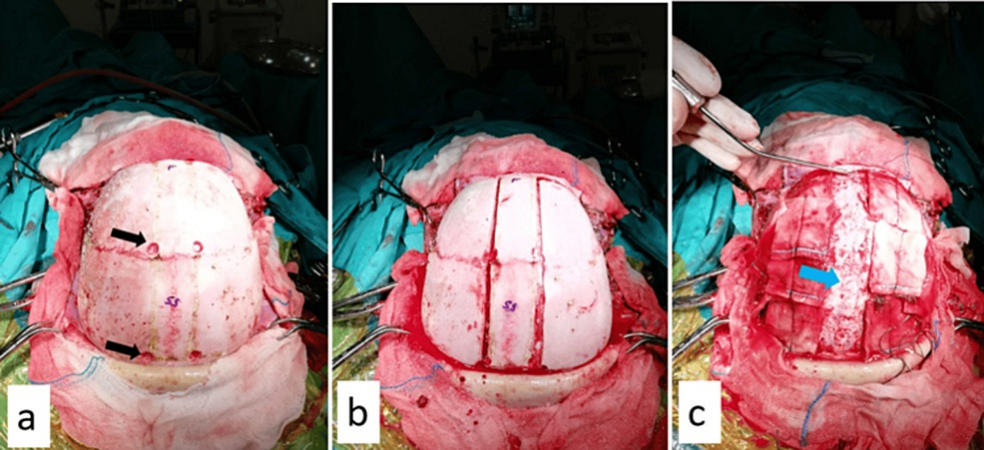
a. Burr holes were planned 1.5 cm lateral to the sagittal suture, both anterior and posterior to the coronary suture. Two anterior burr holes were planned 1 cm anterior to the coronary suture (upper black arrow), and two posterior burr holes were planned 4 cm posterior to the coronary suture (lower black arrow). b. All burr hole sites were connected epidurally, and the skull cap was removed with the last cut across the superior sagittal sinus. c. The bone graft was removed bilaterally. The superior sagittal sinus is pointed (blue arrow).

**Figure 6:**
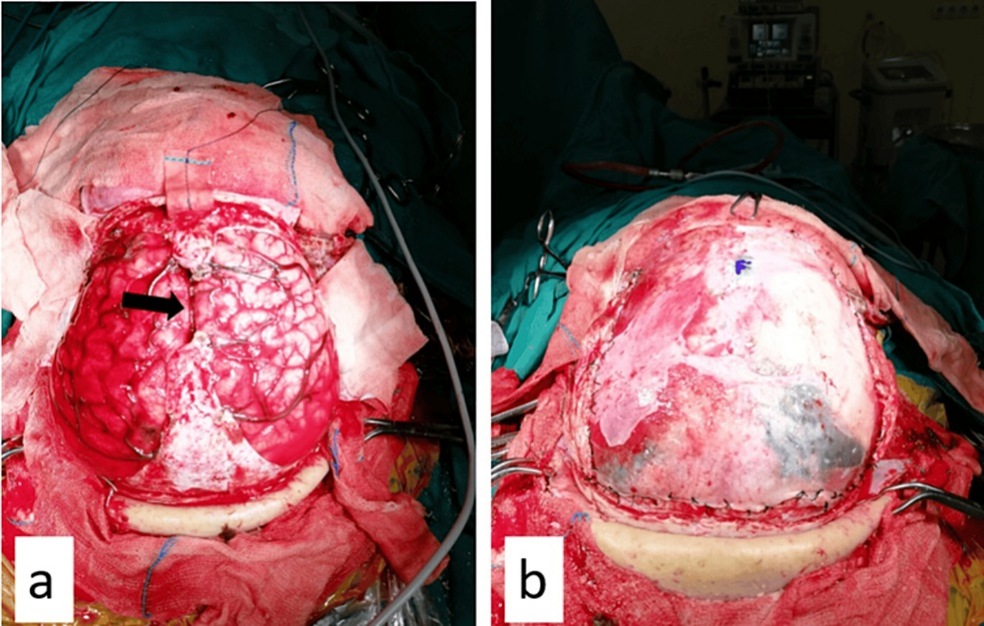
a. After bilateral opening of the dura mater, the superior sagittal sinus was ligated 2 cm anterior to the coronary suture and cut after coagulation with bipolar (black arrow), and the falx was cut 2 cm deeper at the same level. b. Duraplasty with a frontal-based galeal graft was initially used for duraplasty.

The Kjellberg approach is defined as bilateral frontotemporal decompression. As our patient was three years old, had a GCS of four, and diffuse edema, the Kjellberg approach was modified, and bilateral frontotemporoparietal decompression was performed (Figures 3-6).

## Discussion

We report the surgical results of a three-year-old male patient who was brought to the emergency department after a car accident. A bilateral decompressive craniectomy was performed for severe traumatic brain injury. The patient, who underwent replacement cranioplasty in the second postoperative month, was followed up with a GCS of 15 and no neurological deficits.

Decompressive craniectomy remains a controversial topic among neurosurgeons. There is no consensus on whether or not to operate in cases of severe TBI [11]. Decompressive craniectomy in children with severe TBI is associated with good neurological outcomes, according to the results of a retrospective multicentre study in a large paediatric population [9].

There is an inverse relationship between the size of the craniectomy area and mortality in decompression surgery. Mortality is especially high in cases with craniectomies smaller than 10 cm [12]. Stiver stated that the reason for this may be the stretching of axons because of herniation of brain tissue from the decompression area [13]. Jo et al. reported that the results were better in patients with a larger decompression area [14]. Large decompression can be applied as in our case to prevent axonal damage (Figure 2).

The most important prognostic factor in decompressive craniectomy surgery is age [15-17]. Polin et al. performed a modified Kjellberg procedure in 35 patients and reported that the results were better in paediatric patients. At the same time, it was reported that surgery performed 48 hours after trauma and ICP (intracranial pressure) value not remaining above 40 for a long time favourably affected the prognosis [18]. Our patient was also a paediatric patient and was taken to surgery during the third hour of trauma. We think that this may be the reason for early neurological recovery.

Moskowitz et al. investigated prognostic factors in 112 patients who underwent decompressive craniectomy and reported that advanced age, postoperative hypernatremia, and posttraumatic hydrocephalus were poor prognostic factors [17]. Postoperative hypernatremia and postoperative hydrocephalus did not develop in our patient.

Subdural collections and hydrocephalus have been reported to be the most common complications following decompressive craniectomy [19]. In our case, hydrocephalus did not develop, but subdural hygroma developed in the late postoperative period and resolved spontaneously at the second-month follow-up after cranioplasty. Yang et al. reported that the complication rate was higher in elderly patients in a series of 108 cases [20]. No complication other than subdural hygroma was observed in our patient.

## Conclusions

No study has sufficiently established the advantages of decompressive craniectomy in children with severe TBI. It is not possible to draw conclusions about the efficacy of an individual case intervention. However, a patient with a low GCS demonstrated positive neurological improvement following extensive decompression. Numerous authors have reported favourable neurological outcomes with decompressive craniectomy in paediatric patients suffering from severe TBI. For successful decompression, it is imperative to open the dura and perform a wide craniectomy.
